# Combined Amplification and Sound Generation for Tinnitus: A Scoping Review

**DOI:** 10.1097/AUD.0000000000000516

**Published:** 2018-04-27

**Authors:** Lindsey Tutaj, Derek J. Hoare, Magdalena Sereda

**Affiliations:** 1 Department of Audiology, School of Allied Health Sciences, Faculty of Health and Life Sciences, De Montfort University, Leicester, United Kingdom; 2 NIHR Nottingham Biomedical Research Centre, Nottingham, United Kingdom; 3 Otology and Hearing Group, Division of Clinical Neuroscience, School of Medicine, University of Nottingham, United Kingdom.

**Keywords:** Amplification, Combination hearing aids, Masking, Sound generation, Sound therapy, Tinnitus, Wireless streaming

## Abstract

**Objectives:**

In most cases, tinnitus is accompanied by some degree of hearing loss. Current tinnitus management guidelines recognize the importance of addressing hearing difficulties, with hearing aids being a common option. Sound therapy is the preferred mode of audiological tinnitus management in many countries, including in the United Kingdom. Combination instruments provide a further option for those with an aidable hearing loss, as they combine amplification with a sound generation option. The aims of this scoping review were to catalog the existing body of evidence on combined amplification and sound generation for tinnitus and consider opportunities for further research or evidence synthesis.

**Design:**

A scoping review is a rigorous way to identify and review an established body of knowledge in the field for suggestive but not definitive findings and gaps in current knowledge. A wide variety of databases were used to ensure that all relevant records within the scope of this review were captured, including gray literature, conference proceedings, dissertations and theses, and peer-reviewed articles. Data were gathered using scoping review methodology and consisted of the following steps: (1) identifying potentially relevant records; (2) selecting relevant records; (3) extracting data; and (4) collating, summarizing, and reporting results.

**Results:**

Searches using 20 different databases covered peer-reviewed and gray literature and returned 5959 records. After exclusion of duplicates and works that were out of scope, 89 records remained for further analysis. A large number of records identified varied considerably in methodology, applied management programs, and type of devices. There were significant differences in practice between different countries and clinics regarding candidature and fitting of combination aids, partly driven by the application of different management programs.

**Conclusions:**

Further studies on the use and effects of combined amplification and sound generation for tinnitus are indicated, including further efficacy studies, evidence synthesis, development of guidelines, and recommended procedures that are based on existing knowledge, expert knowledge, and clinical service evaluations.

## Abstract 2

Supplemental Digital Content is available in the text.

## INTRODUCTION

In most cases, tinnitus is accompanied by some degree of hearing loss (Shargorodsky et al. 2010). Current tinnitus management guidelines (Tunkel et al. 2014) recognize the importance of addressing hearing difficulties, with hearing aids being a common option (Hoare et al. 2014). Some studies estimate that up to 90% of people with tinnitus might benefit from the amplification (Johnson 1998; Schechter & Henry 2002). Sound therapy is the preferred mode of audiological tinnitus management in many countries, including in the United Kingdom, and refers to a wearable sound generator or hearing aid (Hobson et al. 2012). Postulated mechanisms through which sound therapy can be beneficial for tinnitus include reducing tinnitus intrusiveness, aiding habituation, distracting attention from tinnitus, and triggering neuroplasticity within the brain (Newman & Sandridge 2012). Combined amplification and sound generation in the form of combination hearing aids or wireless streaming provide a further option for those with an aidable hearing loss. Combination hearing aids combine amplification with a sound generation option within one device, and new generations of such devices offer the same quality of amplification as “standard” hearing aids (Henry et al. 2004). Recent developments in technology have given rise to manufacturers incorporating a wireless streaming option into their devices. Wireless streaming allows any sound that might be beneficial in managing patients’ tinnitus to be streamed into their hearing aids.

Hobson et al (2012) conducted a Cochrane review of sound therapy (described as masking) in the management of tinnitus, and four of their included studies used combination aids as one of the interventions (Mehlum et al. 1984; Hazell et al. 1985; Henry et al. 2006a, b). The conclusion of that review was that there was no evidence for a significant change in loudness or severity of tinnitus when sound-generating devices were used as a sole intervention. However, there were also no adverse events associated with sound therapy, and the interventions were found to be safe. Therefore, authors concluded that the lack of evidence should not preclude the use of noise-generating devices (including combination aids) in tinnitus management.

Tunkel et al. (2014) stated that clinicians might recommend sound therapy to patients with persistent, bothersome tinnitus. However, sound therapy was presented only as an option as the strength of evidence for its effectiveness was low. Tunkel et al. (2014) listed combined amplification and sound generation as one of the options for sound therapy and did not make detailed recommendations about candidacy and fitting. They stated, however, that patient preferences should play a significant role in deciding whether to pursue sound therapy and in choosing the particular option. In the United Kingdom, the Department of Health Good Practice Guide (2009) recommended making sound therapy available for patients with tinnitus, but it lacked any recommendations about candidature and prescription options for combined amplification and sound generation. Nor did it specify the acoustic features of the sounds being recommended. The Tinnitus Research Initiative algorithm suggested using combination hearing aids “for intrusive tinnitus where hearing aids alone are ineffective” (Biesinger et al. 2011). This recommendation was not evidence based, nor did it advise on hearing loss characteristics or device prescription options.

Historically, sound was used to mask tinnitus, that is, reduce tinnitus loudness or make tinnitus inaudible (Hoare et al. 2014). In recent years, rather than talking exclusively about maskers, clinicians and researchers would rather use the term sound generators, as masking of the tinnitus percept would not be the only goal and mechanism of action when it comes to sound therapy. Henry et al. (2004, 2008a) applied the definition of tinnitus relief as reduction in annoyance caused by tinnitus, regardless of the mechanism by which it was achieved (masking, partial masking, or not masking the tinnitus). However, even sounds that do not mask tinnitus could provide relief by aiding relaxation (soothing sounds) or providing distraction from tinnitus (interesting sounds; Henry et al. 2008a). To date, there has not been a comprehensive review of what sounds would be offered or recommended by clinicians and how they would be used in everyday situations by patients.

The aim of the scoping review was to map relevant literature in the topic of interest (Arksey & O’Malley 2005). This type of literature review is a rigorous way to identify and review an established body of knowledge in the field for suggestive but not definitive findings and gaps in current knowledge (Arksey & O’Malley 2005; Levac et al. 2010). The primary aim of this scoping review was to catalog the existing body of knowledge on combined amplification and sound generation for tinnitus; who was fitted; what sounds were used/recommended. We focused on records where combined amplification and sound generation were used as a singular treatment (i.e., main focus was on sound therapy with a minimal educational/counseling component).

Secondary aims of the review were to describe (1) the literature where combined amplification and sound generation were used as part of a treatment program, and (2) where research gaps (opportunities for research) or large bodies of evidence already existed (opportunities for evidence synthesis).

## MATERIALS AND METHODS

We followed Arksey and O’Malley (2005) methodological framework recommendations. The procedure consisted of the following steps: (1) identifying potentially relevant records; (2) selecting relevant records; (3) extracting data items; and (4) collating, summarizing, and reporting the results. Step 4 involved grouping results together according to their main findings and themes (Boyatzis 1998).

### Step 1: Identifying Potentially Relevant Records

A wide variety of databases were used to ensure that all relevant records within the scope of this review were captured. This included gray literature, conference proceedings, dissertations and theses, as well as peer-reviewed journal articles. Search engines used were MEDLINE, Web of Science, The Cumulative Index to Nursing and Allied Health Literature (CINAHL), EMBASE, PsycINFO, the Cochrane Central Register of Controlled Trials (CENTRAL), Latin American and Caribbean Health Sciences Literature (LILACS), KoreaMed, IndMed, PakMediNet, CAB Abstracts, Clinicaltrials.gov, www.who.int/trialsearch, Google Scholar, China National Knowledge Infrastructure (CNKI), International Standard Randomized Controlled Trial Number (ISRCTN), International Clinical Trials Registry Platform (IC-TRP), DART Europe (UK and European), ProQuest Dissertations and Theses (US), Cos Conference Papers, Google Scholar, Institute of Electrical and Electronics Engineers and Institution of Engineering and Technology (IEEE/IET) Electronic Library, Scopus, Web of Science, Zetoc, Open Gray. Gray literature records were included if the full text was accessible (conference proceeding, website). Manual searches of key hearing aid manufacturers’ websites were performed to look for further journal publications. A hand search of reference lists from included articles was performed after final inclusion, last updated in May 2017.

**Figure 1.**
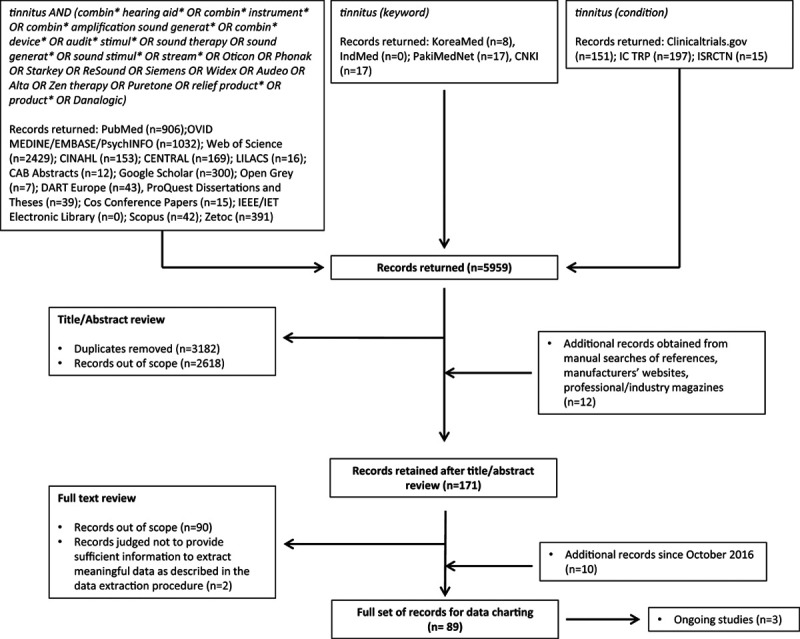
Flow diagram illustrating search strategy and scoping review stages.

The search strategy applied to each search engine is reported in Figure 1. Where possible, the full search strategy was applied. However, it was not always possible to apply the full search strategy to all databases as some do not have that option. For those databases, “tinnitus” was searched as a keyword. For searches of clinical trial databases, the term tinnitus was searched for as the condition. There was no restriction imposed on year, study design, or language. Records in other languages were translated into English.

The search of Google Scholar returned many thousands of records. Therefore, as this particular search engine arranges the results by relevance, all records were included up to the point when there were no more relevant records identified on three consecutive pages of 10 records. Three hundred records (corresponding to the first 30 pages) were carried forward for title/abstract screening. These search strategies returned 5959 records altogether.

### Step 2: Selecting Relevant Records

The records were included if they involved combined amplification (combination aids or wireless streaming) as a treatment option. Duplicate (n = 3182) records were removed. The next step considered title and abstracts, and a further 2618 records were judged to be out of scope and were excluded. All excluded records involved forms of therapy that were not combined amplification and sound generation.

Record screening was completed independently by two authors to avoid any bias (Levac et al. 2010). Two authors conducted the selection process, and agreement was reached for the “out of scope” records. One hundred seventy-one records were retrieved for full text review (Fig. 1).

A further 90 records were deemed out of scope, and two records did not provide meaningful data to be extracted. Therefore, from the 171 records, a further 92 records were excluded, leaving 79. A further 10 records were identified in update searches, giving a total of 89 records, which were retained for data extraction.

### Step 3: Extracting Data Items

A template for data extraction was created and agreed by two authors. The data extraction form was piloted using two records that were excluded as they regarded another type of intervention (not combined amplification and sound generation). Data extracted were type of technology used (combination aids of wireless streaming), type of study (e.g., investigational studies, reviews, concept description), context, research question, main findings, additional results relevant to the review questions, general characteristics of the records (year, country, journal title, experimental setting), manufacturer; peer-review (yes/no), study design (prospective/retrospective, design), subject population, number of subjects contributing data, power analysis (yes/no), funding to account for any potential bias, and outcome measures; type of sound used, management program (yes/no, name of the program), candidacy, fitting, instructions to patients.

Two authors conducted data extraction independently. A meeting between the two authors was organized to resolve any discrepancies and agree on a final data set. Most discrepancies involved one or other author identifying additional information relevant to the data items of interest, for example, a potentially relevant detail spotted in a line of discussion that was not identified or extracted by the other author. Where these discrepancies in the extracted data were present, authors referred to the original record and agreed a final selection of data through discussion.

### Step 4: Cataloging the Results

To provide a structure for subsequent content analysis and narrative review, records were categorized according to whether combination hearing aids were used as a primary treatment or were used as part of a treatment program or package. There was a possibility to assign the same record to more than one category if authors decided that one would not reflect the content adequately. Thematic analysis was conducted to describe the main findings of the records grouped in broad themes.

## RESULTS

### Existing Body of Knowledge on Combined Amplification and Sound Generation for Tinnitus

The majority of records within scope of the research question comprised literature reviews, guides, or practice/concept descriptions (n = 48), followed by investigational studies (n = 38). Furthermore, the majority of records were published in peer-reviewed journals (n = 64). Eighty-three records regarded combination hearing aids, while only six records regarded wireless streaming. Methodology for investigational studies varied considerably with a majority of uncontrolled before-and-after studies. Altogether, 35 investigational studies applied the prospective design. Sample size varied from one to 1888, with only one study (dos Santos et al. 2014) basing sample size on a power analysis.

One potential source of bias was whether full or part funding for the study or devices was provided by manufacturers of hearing aids, or where there were authors who were employed by the manufacturer of investigated devices. Such conflict of interest was reported in 17 included records.

### Evolution Over Time and Country of Origin

**Figure 2.**
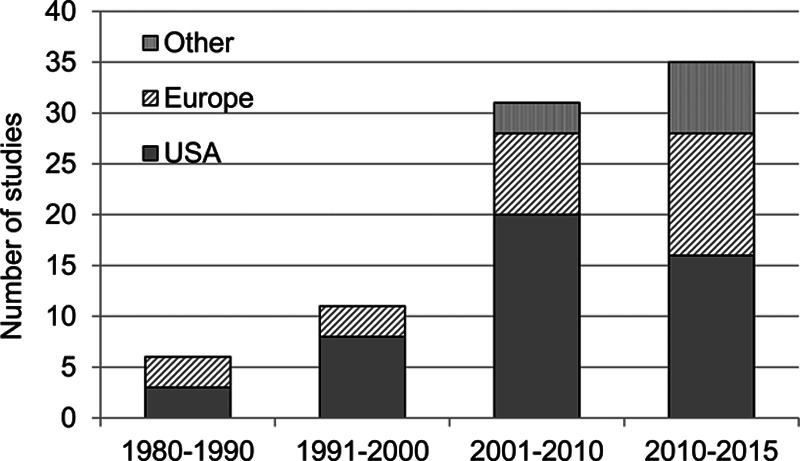
Distribution of included records over time.

The distribution of records over time reflected the introduction into clinical practice and recent increased use of combination hearing aids as a management option, a trend likely related to the improved quality of amplification provided by the instruments. Records came from the United States (n = 48), followed by the United Kingdom (n = 16), Germany (n = 5), Brazil (n = 3), Italy (n = 3), and Australia, China, Denmark, France, Greece, Japan, Mexico, Spain, Switzerland, Turkey, and New Zealand (n = 1 in each country; Fig. 2). Note that the proportion of studies conducted in the countries other than the United States increased over the years.

### Outcome Measures to Assess Clinical Efficacy and “Therapeutic Benefit”

**Figure 3.**
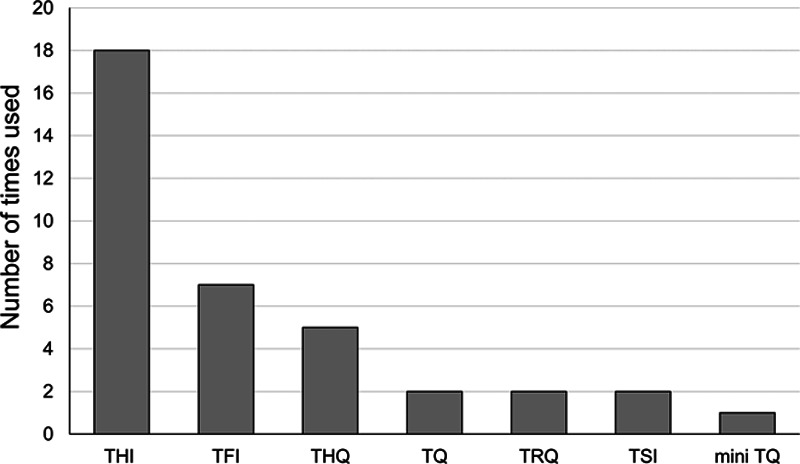
Tinnitus-specific questionnaires used to measure efficacy in the included studies. TFI, Tinnitus Functional Index; THI, Tinnitus Handicap Inventory; THQ, Tinnitus Handicap Questionnaire; TQ, Tinnitus Questionnaire; TRQ, Tinnitus Reaction Questionnaire; TSI, Tinnitus Severity Index.

**Figure 4.**
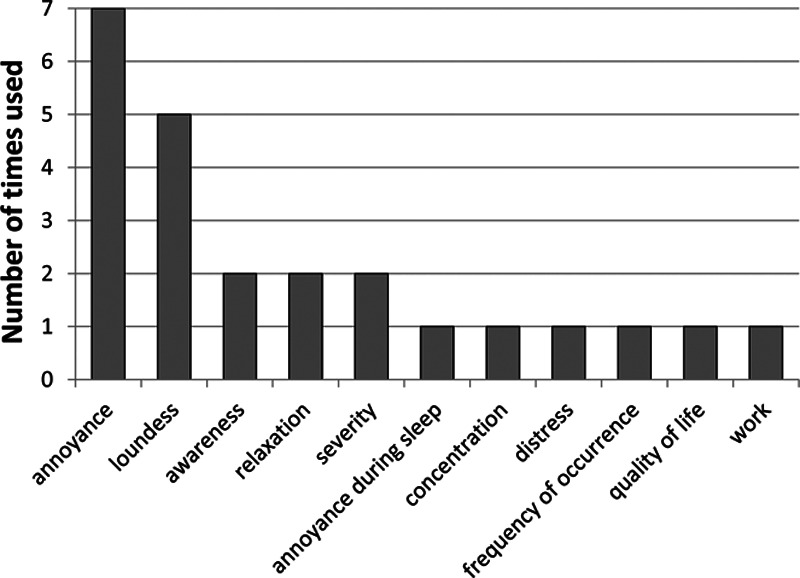
Visual/numeric analog scales used to measure efficacy in the included studies.

Forty-four different outcome measures were identified in the included studies. Most commonly used were tinnitus-specific questionnaires (n = 37, across 28 studies) followed by Visual/Numeric Analog Scales (n = 25, across 12 studies). Among the tinnitus-specific questionnaires used, the most common were Tinnitus Handicap Inventory (THI, n = 18), Tinnitus Functional Index (n = 7), and Tinnitus Handicap Questionnaire (THQ; n = 5; Fig. 3). The feature most commonly measured with Visual/Numeric Analog Scales was tinnitus annoyance (n = 7), followed by tinnitus loudness (n = 5; Fig. 4). Other measures included interview (n = 3) and number of patients purchasing the devices (n = 3).

### Records Where Combined Amplification and Sound Generation Are Discussed or Evaluated as a Primary Treatment

Sixty records described combined amplification and sound generation as a primary treatment. See Table 1, Supplemental Digital Content 1, http://links.lww.com/EANDH/A390 for the full list and characteristics of the records. Investigational studies (n = 28) showed a large variability in the study design, with uncontrolled before-and-after design being the most common (n = 11), followed by randomized or quasi-randomized trials (n = 8) and crossover studies (n = 3). Other study designs included historically controlled trial (n = 1), nonrandomized controlled study (n = 1), case reports (n = 2), a pilot study (n = 1) and a survey of clinicians (n = 1). There were 24 reviews in this category, of which four were systematic reviews including one Cochrane review (Hobson et al. 2012). The remaining records were practice guide/descriptions (n = 5) or concept/product descriptions (n = 3). Fifty-seven studies in this category regarded combination hearing aids only, two regarded combination hearing aids and wireless streaming, while one compared the two options.

Efficacy was the main research topic in 34 records. Twenty-three records described combined amplification and sound generation as a management option, two presented methods to determine the candidacy for combination hearing aids, and one focused on patient satisfaction with combination aids.

From 34 studies where the main research question related to the efficacy of combined amplification and sound generation, only eight studies compared combination aids with amplification only. Among those were four small-scale randomized controlled trials (RCTs) (Stephens & Corcoran 1985; dos Santos et al. 2014; Hicks et al. 2014; Henry et al. 2015), two prospective crossover before-and-after studies (Mehlum et al. 1984; Frachet et al. 2004), one prospective controlled study (Hazell et al. 1985), and one pilot study (dos Santos et al. 2012). Seven of the studies were published in peer-reviewed journals, and one was a conference abstract (dos Santos et al. 2012). Those studies typically reported equal effects of combination aids and amplification only. Before-and-after studies with no control condition, which constituted the majority of research studies that investigated efficacy of combined amplification and sound generation, noted reductions in tinnitus annoyance or distress, or improvement in quality of life, after the intervention. Eight out of nine literature reviews concluded a lack of evidence for efficacy of combination aids or sound therapy, in general, in the management of tinnitus. Only one review (Sweetow 2013) concluded that combination aids were effective in promoting relaxation and reducing annoyance from tinnitus. That review, however, concentrated on the effects of fractal tones in the management of tinnitus, which was not a standard option in many tinnitus clinics. It is also worth noting that the studies included in the review by Sweetow (2013) were before-and-after studies; therefore, no comparison with the effects of amplification or other management options was possible. There was a necessary overlap in content between reviews and investigational studies of efficacy as reviews were based on the records included in the current scoping review. Two records that compared efficacy of different noise options (Barozzi et al. 2016; Searchfield et al. 2016) obtained equal improvement in tinnitus, regardless of the applied sound option.

Records that described combination aids as a management option but did not report investigative studies were literature reviews (n = 15), practice guides (n = 4), concept/product descriptions (n = 3), and a patient survey exploring their characteristics (n = 1). The records often briefly mentioned combination aids as a management option without providing any further details. Most of these records provided recommendations regarding candidacy for combination aids, with some records providing more detail on available noise options and fitting recommendations. Two records described wireless streaming as a flexible option that addressed the needs of different tinnitus management plans (Piskosz 2012; Piskosz & Dyrlund 2015).

Two records focused on candidacy and choice of devices, including combination aids for tinnitus. Newman and Sandridge (2006) introduced the Sound Therapy Option Profile, which was developed as a guiding tool for selection of devices used in tinnitus treatment. Sound Therapy Option Profile comprises 11 items and based on the patient’s answers indicates which device would be the optimal choice for the patient. Schechter and Henry (2002) discussed selection of ear level devices used in Tinnitus Masking (TM) program for veterans.

One record investigated patient satisfaction with Widex Clear hearing aids (Häberle & Hoejgaard Kristensen 2012), concluding that incorporating sound therapy programs in hearing aids might persuade people with hearing loss and tinnitus to seek treatment.

### Candidacy

Combination aids would be offered in principle to those who have tinnitus and co-existing hearing loss, with several studies indicating clinically significant, bothersome, or debilitating tinnitus as an additional criterion (Gabriels 2001; Frachet et al. 2004; Henry et al. 2005, 2006a; Fioretti et al. 2012; Hicks et al. 2014; Hoare et al. 2014; Johansen et al. 2014). Some authors specified that tinnitus should be a primary complaint (Stephens & Corcoran 1985; Sweetow & Sabes 2010). Several records described combination devices in the context of TM (Vernon & Meikle 2000; Henry et al. 2002a, b; Schechter & Henry 2002;). The main objective of TM is to provide immediate relief from tinnitus through the use of ear-level devices (noise generators, hearing aids, or combination hearing aids). In the studies reviewed, the choice of device was decided by trialing all possible options and having the patient choose the combination that provided maximum relief. Some authors advised trying amplification only first and adding the noise if amplification only was not sufficient in decreasing tinnitus annoyance (Piskosz 2012), or tinnitus was still interfering with the daily life (Hall 2013), or did not provide sufficient masking (Johnson et al. 1989; Vernon & Meikle 2000; Rosanowski et al. 2001; Schechter & Henry 2002; Piskosz 2012). To achieve masking and provide high-frequency stimulation, Vernon and Meikle (2000, 2003) suggested that combination aids should be offered to patients with high-pitched tinnitus and high-frequency hearing loss (above 4 kHz) as normal environmental sounds are usually limited to frequencies below 4 kHz. Some additional criteria mentioned were motivation to use and comply with the intervention (Henry et al. 2006a; Häberle & Hoejgaard Kristensen 2012; Newman & Sandridge 2012), minimum masking levels or “maskability” (Goldstein & Shulman 1996; Peifer et al. 1999), difficulty seeping (Johnson et al. 1989), and cost (Newman & Sandridge 2012). For the research studies, additional inclusion criteria were often specified such as duration of tinnitus (Frachet et al. 2004; dos Santos et al. 2014; Barozzi et al. 2016), degree of hearing loss (Piskosz & Kulkarni 2010; dos Santos et al. 2012; dos Santos et al. 2014; Henry et al. 2015; Searchfield et al. 2016; Berberian et al. 2017), perceived hearing difficulties (Henry et al. 2015), laterality of hearing loss (Henry et al. 2015; Searchfield et al. 2016; Berberian et al. 2017), or recent hearing aid experience (dos Santos et al. 2014; Johansen et al. 2014; Henry et al. 2015). One study (Sereda et al. 2017) recruited existing combination aids users.

### Choice of Sounds

TM permits use of any sound that provides maximum masking benefit (Henry et al. 2002); therefore, the choice of sound is usually based on a combination of effectiveness and acceptability. Records that did not follow the TM program more often listed broadband noise (n = 14) or fractal (Zen) tones (n = 9). Two records referred to nature sounds (Barozzi et al. 2016; Sereda et al. 2017), three referred to broadband noise shape according to individual audiogram (Stephens & Corcoran 1985; Henry et al. 2015; Williams & Patel 2016), one referred to narrowband signal (Tóth et al. 2014), and one referred to narrowband signal focused on the frequency of tinnitus (Hoare et al. 2014). Additional adjustments to the noise included amplitude modulation (Piskosz & Kulkarni 2010; Smith et al. 2013; Hoare et al. 2014; Henry et al. 2015), frequency shaping (Piskosz & Kulkarni 2010; Fioretti et al. 2012; Smith et al. 2013), or tempo and pitch adjustments to the fractal tones (Sweetow & Sabes 2010).

Wireless streaming allows any sound to be transmitted through to the hearing aids. Piskosz & Dyrlund (2015) described wireless streaming as an option for those who prefer not to listen to a broadband noise and would benefit from a wider selection of sounds. Sounds mentioned in the context of wireless streaming included pink, red, blue, violet, nature sounds, environmental sounds, appliances, fans, music, and speech (Piskosz 2012; Piskosz & Dyrlund 2015; Barozzi et al. 2016). The sounds could also be personalized by simultaneous layering of up to five sounds and adjusting volume of each sound independently using sound mixer (Piskosz & Dyrlund 2015).

### Level of Masking Sound

Vernon and Meikle (2000) describes how within TM use of either complete (completely covering patient’s tinnitus) or partial (reducing the perceived loudness of tinnitus) masking when fitting combination aids is permitted because the main goal is to provide immediate relief from tinnitus. The level of noise is chosen by the patient, with the caveat that the level of noise should be tolerable and not louder than necessary (Henry et al. 2006a, b).

In the included records that did not follow the TM approach, the level of noise was advised to be set at the mixing point (the level where the tinnitus sound and the sound generator stimulus start to blend together; Frachet et al. 2004; McFerran & Phillips 2007; Fioretti et al. 2012; Barozzi et al. 2016), at the lowest level providing tinnitus relief, no higher than is required to mask tinnitus (Hazell 1990; Goldstein & Shulman 1992; Vernon & Meikle 2000; Schechter & Henry 2002; dos Santos et al. 2014; Tóth et al. 2014), or at a soft level audible to the patient but lower than the level of tinnitus (Hazell 1990; Sweetow & Sabes 2010).

Several records mentioned volume control to adjust the level of the noise (Peifer et al. 1999; Piskosz & Kulkarni 2010; Sereda et al. 2017) or environmental steering option (Piskosz & Kulkarni 2010; Sereda et al. 2017).

Two records described different protocol for sound stimulation in which the masker level is decreased from immediate relief to a background sound (López-González & López-Fernández 2004; Henry et al. 2005). Sequential Sound Therapy (SST) (López-González & López-Fernández 2004) used different levels of masking noise in a sequential manner. The therapy starts with noise set to provide total masking (white noise that abolishes the tinnitus percept) for the first month of adaptation, then of equal loudness to tinnitus for the second month, and finally white noise was set at the loudness below tinnitus for the third and subsequent months of adaptation. Henry et al. (2005) described a progression from “complete masking” to “partial masking” and finally to “non-masking background sound.”

Williams and Patel (2016) commented that the disadvantage of wireless streaming was that the signals could not be customized by the clinician, meaning they are less consistent with the patient’s auditory profile.

### Recommended Daily Use and Adjustments During the Day

Recommended daily use varied widely across records. TM patients were not required to use the devices throughout the day but rather to use them “as needed” and adjustments were allowed (Henry et al. 2006b). Dos Santos et al. (2014) recommended use of the devices for at least 8 hours/day, Sereda et al. (2017) recommended use for at least 6 hours/day. Stephens and Corcoran (1985) recommended using a “masker” for at least 1 hour per day, and López-González and López-Fernández (2004) recommended using the masking noise for 6 hours/day initially and then as needed (or about 2 hours/day). Sweetow and Sabes (2010) recommended using different programs in a variety of everyday situations, and two records recommended using the devices during sleep (Johnson et al. 1989; Vernon & Meikle 2000).

### Laterality of Fitting

TM allows binaural or monaural fitting as long as maximum masking benefit is obtained (Schechter & Henry 2002; Henry et al. 2006a). Bilateral and unilateral fittings were described depending on the study, with varying criteria for choosing bi- vs unilateral combination aids.

For unilateral fitting, laterality depended on the laterality of tinnitus, with unilateral tinnitus being fitted with one combination aid and bilateral tinnitus with two (Mehlum et al. 1984; Hazell et al. 1985; Stephens and Corcoran 1985; Hazell 1990; López-González & López-Fernández 2004), or laterality of hearing loss, with bilateral hearing loss requiring bilateral fitting while unilateral loss requiring unilateral fitting (Stephens & Corcoran 1985; Sweetow & Sabes 2010; Fioretti et al. 2012). López-González & López-Fernández (2004) noted that unilateral tinnitus may shift to the unmasked ear if unilateral fitting was applied, and that in those cases, a second device was indicated. This was observed in 14 out of 26 patients. Moreover, Vernon and Meikle (2000) stated that for patients with bilateral tinnitus, it could not be assumed that both ears required the same type of device as tinnitus could behave differently in two ears. In general, authors suggested that bilateral tinnitus or tinnitus perceived “in the head” most likely required bilateral devices (Hazell et al. 1985; Hazell 1990; Vernon & Meikle 2000). However, they also suggested that in some cases, it was possible to mask bilateral tinnitus with a single device (Hazell 1990), and it was not possible to determine which configuration would work best without a trial (Tyler et al. 1992). Hazell (1990) described two different approaches to bilateral fitting that were used in his clinic. The first involved fitting a single instrument initially and fitting a second instrument once the patient gained confidence in handling the first. The second approach was to fit two instruments at the same time. Hazell (1990) commented that neither of the approaches proved to be better than the other, with both providing satisfactory results.

### Emerging Approaches

Three records presented novel approaches to combined amplification and sound generation. Barozzi et al. (2016) directly compared the efficacy of “nature” and “technical sounds” for tinnitus. Nature sounds were streamed wirelessly to participants’ hearing aids, while “technical sound” was a conventional broadband noise available on commercially available combination aids. Authors concluded that both approaches were effective in improving patients’ coping with tinnitus.

In another approach, Searchfield et al. (2016) compared customized spatial (3D) masking (novel approach) to conventional bilateral masking. Spatial masking allowed presentation of masking at the same location in a 3D auditory space as tinnitus. Searchfield et al. (2016) hypothesized that this type of stimulus would be more effective than masking not localized to the perceived position of tinnitus. Feasibility and pilot studies were conducted and further trials recommended.

More recently, Hauptmann et al. 2017 described a case study in which Acoustic coordinated reset (CR) therapy was delivered via commercially available combination aids using streamer. Acoustic CR neuromodulation used sequences of acoustic tonal stimuli above and below the tinnitus frequency and aimed to reduce pathological synchronous activity in the brain presumed to be tinnitus generating. Previous to that feasibility study, acoustic CR neuromodulation was delivered using an mp3-like device via headphones.

### Records Where Combined Amplification and Sound Generation Are Discussed as Part of a Treatment With Other Components

Thirty-eight records described combined amplification and sound generation as a part of a multicomponent treatment, including Tinnitus Retraining Therapy (TRT, n = 29), Widex Zen Therapy (n = 7), drugs and instrumentation (n = 2), Progressive Tinnitus Management (PTM, n = 2), Tinnitus Activities Treatment (TAT, n = 1), and multidisciplinary tinnitus management (n = 1). See Table 2, Supplemental Digital Content 2, http://links.lww.com/EANDH/A391 for the full list and characteristics of the records. Among those were 15 investigational studies. Investigational studies showed a large variability in study design, including uncontrolled before-and-after design (n = 5), nonrandomized controlled studies (n = 4), randomized or quasi-randomized trials (n = 3), a crossover pilot (n = 1), a clinician survey on the use of Zen for tinnitus (n = 1), and a retrospective uncontrolled before-and-after study (n = 1). The other records were literature reviews (n = 16), practice guide/description (n = 5), and concept/product descriptions (n = 2).

### Program Characteristics

Recommendations on candidacy and fitting of combination hearing aids strongly depended on the management program followed. There were also marked differences in approaches between different management programs including fitting laterality and recommended daily use.

In principle, all programs advised the use of combination aids for people who have tinnitus and co-existing hearing loss. However, the candidacy decisions were based on different criteria in each of the programs. For example, within TRT, ear-level devices were recommended for TRT Category 2 patients (severe tinnitus and significant subjective hearing problems). Combination aids were a preferred option, with hearing aids only recommended in cases where cost was an issue or the specifics of hearing loss did not allow for fitting of combination aids (Henry et al. 2002; Jastreboff & Jastreboff 2003). In TRT, binaural fitting of the devices was recommended (Jastreboff & Jastreboff 2000). According to TRT protocol, the level of noise should be adjusted to what the authors termed the “mixing” or “blending” point (Jastreboff 2007; McFerran 2009; Korres et al. 2010) or below that level (Jastreboff & Jastreboff 2006). TRT protocol asserts tinnitus should be heard distinctly, the noise should not cause annoyance, or completely mask the tinnitus. The volume of the noise should not be set too close to hearing threshold to avoid exacerbating tinnitus. Sound adjusted in such a way might have not always been heard, especially in noisy environments (Jastreboff & Jastreboff 2006). TRT requires continued use of the devices throughout the day, and users were asked to “set and forget” their devices (no adjustments during the day are allowed; Henry et al. 2006a, b). In included records, the recommended wear time during the day for TRT varied from 6 to 24 hours (Jastreboff 2007; Korres et al. 2010; Butcher & Davies 2012).

In Zen Therapy, counseling and relaxation play a crucial role in tinnitus management, and combination aids were recommended for anyone with tinnitus as a main complaint and hearing loss (Sweetow & Kragh Jeppesen 2012; Herzfeld et al. 2014; Sweetow et al. 2015). Similarly to TRT, for combination aids used as a part of Zen Therapy, binaural fitting was recommended, with sound set at a soft level, below the level of tinnitus, which was audible to the patient but not loud enough to interfere with comfortable listening or speech intelligibility. The volume was recommended to be set so that the annoyance level of the tinnitus just began to decrease (Sweetow 2013). In Zen Therapy, it is also recommended that the devices are worn during waking hours, but that “frequent volume changes” are avoided (Herzfeld et al. 2014; Sweetow et al. 2015).

Similar to TRT, TAT addressed the reaction to tinnitus and uses informational counseling, activities engagement, sound therapy (Tyler et al. 2007; Powers & dos Santos 2015). In this treatment, sound produced by combination aids should be audible but not achieve mixing point, and comfortable to the patient, such that they use the lowest level of masker that would provide adequate relief (Tyler et al. 2007). For patients with hearing loss in a TAT program, amplification was the first option, and if the patient’s reaction to tinnitus did not improve, then masking sound was added (Powers & dos Santos 2015).

PTM is a five-level approach to management of patient with tinnitus consisting (1) screening for clinically significant tinnitus; (2) group education; (3) intake assessment; (4) application of treatment program if further treatment necessary; and (5) extension and broadening of treatment if results not satisfactory (Henry et al. 2008a, b). Within the PTM protocol, devices are introduced as a treatment option at level 4 for those patients for whom education and counseling were not sufficient. The use of therapeutic sound within PTM (including combination aids) is flexible to address individual preferences and needs, and the main goal of PTM is for patients to learn to develop and implement individualized plans for using sound to manage their tinnitus (Henry et al. 2008a). The three sound strategies within PTM are soothing sounds (to produce sense of relief from tinnitus-associated stress), background sound (passively diverting attention from tinnitus by reducing contrast between environment and tinnitus), and interesting sounds (actively diverting attention away from tinnitus). The audiologist could decide to administer different management program (including TRT, TM, or TAT) taking into account the patient’s individual needs and preferences, and candidacy for the devices would depend on the program followed (Henry et al. 2008a); fitting parameters depend on the management program applied.

Goldstein and Shulman (2010) describe Tinnitus Targeted Therapy as a combined treatment of medication and instrumentation focusing on pharmacotherapy that evolved from authors’ clinical experience. Instrumentation was recommended to 10%–15% of tinnitus patients resistant to pharmacotherapeutic modalities for tinnitus relief. Different types of instruments could be recommended, including combination hearing aids for people with mixed or sensorineural hearing loss and tinnitus. Oz et al. (2013) described use of combination aids (and sound generators) in conjunction with administration of betahistine dihydrochloride (2HCl). However, the combination aid subgroup was not analyzed.

Currently, there are 2 RCTs registered that used combination hearing aids as an intervention (Table 1). Additionally, one Cochrane review protocol was published, which aimed to summarize the evidence from Cochrane systematic reviews on the efficacy and safety of interventions for tinnitus in adults, including TRT and sound therapy (Maldonado Fernández et al. 2015).

**TABLE 1.**
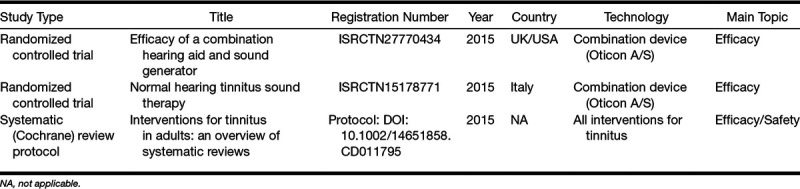
Ongoing studies

## DISCUSSION

This scoping review was undertaken to establish what information was available from the existing literature and identify any gaps and opportunities in the current body of knowledge regarding combined amplification and sound generation for tinnitus.

### Existing Knowledge

Over the years, the number of studies looking at the combined amplification and sound generation for tinnitus increased, reflecting the increased popularity of this management option in clinical practice. Combination aids were used as a part of many different management programs (TRT, TM, Zen Therapy, PTM) and outside of those.

There was a rich literature describing the principles of various management programs and providing guidelines on different aspects of tinnitus management within those programs, with many having strict criteria regarding candidacy, fitting, and use of combined amplification and sound generation by the patient. Most programs (TRT, TM, and PTM) did not specifically recommend the use of certain type of device such as combination hearing aid but suggest that positive results could be achieved using sound therapy in general, regardless of the mode of delivery. All of the above programs consisted of various components from which each was postulated to play an important role in the management of tinnitus. Practices were highly variable, however, with different management programs followed by different clinics.

Most records seemed to support the use of combination hearing aids in tinnitus therapy, reporting improvements in tinnitus distress and handicap. Those results were achieved regardless of the management program followed as studies using sound therapy as a part of different programs reported positive results. Moreover, the small number of studies that directly compared different programs or approaches suggested that each of those could provide a reduction in tinnitus distress (von Wedel & von Wedel 2000; Henry et al. 2006a, b; Tyler et al. 2012).

### Opportunities for Research

Although a large number of records was found (n = 89), only 10 compared combined amplification and sound generation for tinnitus to amplification only. Most studies (n = 7) found no difference in tinnitus distress or handicap between amplification only and combination aids. However, the picture was not clear as only two of those studies were RCTs published in peer-reviewed journals (dos Santos et al. 2014; Henry et al. 2015). Both RCTs found no difference between amplification only and combination aids. It is worth noting that both studies had a small number of participants, and both were testing one type of device only.

Current combination aids offered a wide choice of different noise options (Hoare et al. 2013, 2014). While broadband noise (such as white, pink, red, or brown) was a standard option in most of the devices, with options for modulation or filtering, several manufacturers offered additional options such as noise shaped according to the patients audiogram, noise centered either at or away from the tinnitus frequency, or nature sounds. Moreover, wireless streaming options offered endless possibilities when it comes to choosing the optimal sound for tinnitus therapy, including music, environmental sounds, or even individually modified sounds (Piskosz 2012; Piskosz & Dyrlund 2015; Powers & dos Santos 2015). Despite the availability of numerous options, there is lack of RCTs looking at efficacy of different noise options such as fractal tones, nature sounds, or sounds centered at the tinnitus frequency. There was also a lack of studies directly comparing different noise options in terms of their efficacy for tinnitus.

Although several studies mentioned patients’ preferences as an important factor in choosing certain type of devices for tinnitus therapy, preferences for different type of sounds and their acceptability were rarely investigated. In fact, acceptability and preferences regarding different sound options available within combination hearing aids were investigated only in the context of using different fractal tones within the Zen therapy (Sweetow & Sabes 2010). This was surprising as even such options as low or high band-pass filtering could influence the acceptability of the sound and affect adherence to treatment (Terry & Jones 1986; Henry et al. 2008a; Hoare et al. 2013). Therefore, investigating acceptability alongside efficacy is an important component of future studies of combination aids.

Given that most records described combined amplification and sound generation in the context of larger management program, combining multiple approaches to manage tinnitus, it was often difficult or even not possible to draw conclusions specific to that component of the program. It is, therefore, possible that other components, rather than the devices, might have played a role in the observed improvements in tinnitus distress or handicap. For example, McKinney et al. (1999) compared the efficacy of directive counseling in isolation or in conjunction with different types of devices (maskers, hearing aids, and combination hearing aids) as a part of the TRT. The benefit from wearing any form of instrument in addition to directive counseling was minimal, and the authors concluded that directive counseling appears to be the most important element of TRT. Medical Research Council guidance on evaluating complex interventions, when different components might play a role in the therapy, described process evaluation as an essential part of testing of complex intervention in “assessing fidelity and quality of implementation, clarify causal mechanisms, and identify contextual factors associated with variation in outcomes” (Craig et al. 2008). Further guidelines on carrying out the process evaluation were formulated by Moore et al. (2015). Process evaluation would be the first essential step before any RCTs investigating efficacy of combined amplification and sound generation can be designed and would include (1) capturing how the intervention is delivered and exploring any variability in implementation through service evaluation; (2) formulation of guidelines on delivery of the intervention, in particular, candidacy and fitting, through a consensus process with stakeholders; (3) exploring the mechanisms by which intervention produced a change, using qualitative methods, such as focus groups or interviews with intervention users.

Marked variability was observed in candidacy and fitting of combination aids between different management programs and different studies. It was not clear, however, if any of those approaches yielded better results. Not many studies directly compared different management programs. While Henry et al. (2006a, b) found better results for TRT than TM, both approaches provided benefit for tinnitus. Again, whether combination aids had any specific influence on the above results was unclear. López-González and López-Fernández (2004), on the other hand, found better results with SST than TRT, suggesting that the way of introducing and using sound in tinnitus therapy might have been an important factor. However, no further evaluations of SST were identified.

One of the main differences between management programs related to the level of the noise used in the tinnitus therapy. While TM aims to provide immediate relief from tinnitus, achieved by complete masking if possible without setting the masking sound to an uncomfortable level (Henry et al. 2006a, b), other approaches such as TRT and Zen Therapy recommended setting the noise at mixing point or below the level of tinnitus, arguing that habituation was not possible when tinnitus is completely masked or the perception is markedly changed (Jastreboff & Jastreboff 2000, 2003, 2006; Jastreboff 2007). However, it is not clear whether one approach produced better outcomes than another. Tyler et al. (2012) compared TRT with maskers set at mixing point or total masking and did not find significant differences between the two approaches. They concluded that a focus on a mixing point masking is not required for habituation. It is worth noting, however, that only a limited number of patients benefited from the therapy in either group (3/18 and 6/19) as measured with THQ. On the other hand, a study by von Wedel et al. (1997) found that patients reporting partial masking effects through their aids (hearing aid or noise generator) showed more reduction in tinnitus than those using complete masking effects, as measured with the German version of the TQ (*p* < 0.05). Further studies investigating the role of different noise settings within combination aids in achieving long-term relief from tinnitus are needed.

Sweetow et al. (2015) examined long-term usage pattern of sound therapy and/or amplification (up to 12 months), showing that with time the use of amplification-only program increased and the use of Zen programs decreased. However, from the study’s onset, participants were using multiple programs in everyday situations (amplification only or combination of amplification and different Zen options). The pattern of use of different programs in different listening situations was not explored in the reviewed studies. Understanding those patterns could help in tailoring the options provided by combination aids to individual patient’s needs and aid education and counseling regarding the use of sound for tinnitus therapy.

Only six records described wireless streaming as a management option for tinnitus, and only one study compared the efficacy of this option as compared to built-in sound generators. As streaming is a relatively new concept, further studies of its use in clinical practice, acceptability, and efficacy must be completed.

There are clearly many opportunities for further research in this field. Marked differences in clinical practice identified in this review pose a challenge for investigators devising protocols sufficiently flexible to address different patient groups, device options, and related practices. Assessments of patient subgroups for whom protocols should remain flexible versus those assessments of protocols requiring strict evaluation criteria will require justification and thorough understanding of current clinical practice. This could be achieved by country-wide service evaluation and seeking consensus between clinicians (e.g., using a Delphi technique) regarding candidacy and fitting of combination hearing aids. Qualitative and quantitative data should be collected regarding the utilization of different options on the devices in the real world, acceptability of different noise options, and patients’ preferences. The above issues call for more pragmatic trial design.

### Opportunities for Evidence Synthesis

To make evidence synthesis worthwhile, one has to assure a sufficient number of eligible records exist. Applying the most common inclusion criteria for many systematic reviews, namely that studies need to be prospective studies published in peer-reviewed journals, 8 potentially eligible studies were identified in the included records. From those, 5 studies investigated the efficacy of combination hearing aids for tinnitus and 3 investigated sound therapy using different types of devices (combination aids, hearing aids, or maskers). Although data specific to combination aids were not available in the published version of those 3 records, the original data set could be requested from the authors, as is common practice when conducting systematic reviews.

Variability in outcome measures used in clinical trials assessing clinical efficacy of treatment for tinnitus is a recognized problem, and efforts to create core outcome set in tinnitus to be used in clinical trials worldwide are ongoing. A review by Hall et al. (2016) describes the large number and variability in outcome measures used in tinnitus trials. This could pose a challenge when pooling the results of studies together and performing meta-analysis. However, all studies identified as potentially eligible for inclusion in an efficacy review used a tinnitus-specific questionnaire (i.e., Tinnitus Functional Index, THI, Mini TQ, TRQ, and THQ) as one of the outcome measures, with THI used in 5 studies.

No peer-reviewed studies investigating efficacy of wireless streaming were identified.

## CONCLUSIONS

The current review cataloged existing knowledge and knowledge gaps and opportunities around combined amplification and sound generation for tinnitus. A large number of records identified varied considerably in methodology, applied management programs, and type of devices. To inform evidence-based practice, further studies looking at efficacy (clinical trials, evidence synthesis), practice (service evaluation, recommended procedures) acceptability, and preferences (e.g., of wireless streaming) should be conducted.

## ACKNOWLEDGMENTS

This report is independent research by the National Institute for Health Research Biomedical Research Centre Funding Scheme. The views expressed in this publication are those of the author(s) and not necessarily those of the NHS, the National Institute for Health Research, or the Department of Health.

## Supplementary Material
