# Supplementary material for: Combined Amplification and Sound Generation for Tinnitus: A Scoping Review
Source: Ear Hear. 2018 Apr 27;39(3):412–22. doi: 10.1097/AUD.0000000000000516 (PMC7664457; doi:10.1097/AUD.0000000000000516)
Supplement: Supplementary file 1 [file aud-39-412-s001.docx]

| Table. 1 . Charting the data for studies where sound therapy is a main component of the intervention. Records marked with * describe combination aids both as main intervention component as well as in the context of comprehensive intervention strategy (i.e. in conjunction with psychological therapy). For those records the data presented regard only sound therapy as a main component of the intervention. For the data regarding combination aids in the context of comprehensive sound therapy please see Table.2. | | | | | | | | | |
| --- | --- | --- | --- | --- | --- | --- | --- | --- | --- |
| **No** | **Authors, Year** | **Title** | **Participants** | **Type of study** | **Measures** | | **Candidacy** | **Main finding/ Conclusion** | |
| 1 | Hauptmann et al., 2017 | Technical Feasibility of Acoustic Coordinated Reset Therapy for  Tinnitus Delivered via Hearing Aids: A Case Study | 1 | Case report: CR Neuromodulation Therapy delivered via commercially available combination aid using streamer | Tinnitus Handicap Inventory, Visual Analogue Scale (Loudness), Visual Analogue Scale (Annoyance) | | One participant with tinnitus and hearing loss. | It is feasible to deliver the therapy via hearing aids. | |
| 2 | Ibarra et al., 2017* | In-ear medical devices for acoustic therapies in tinnitus treatments, state of the art | NA | Literature review: Combination aids mentioned as a management option. | NA | | Not specified | None specific to combination aids | |
| 3 | Sereda et al., 2017 | Pre-market version of a commercially available hearing instrument with a tinnitus sound  generator: feasibility of evaluation in a clinical trial | 8 | Uncontrolled before and after study/Technical report | Authors’ own questionnaires collected information about acceptability  and preferences of different masker sound options and  patient and audiologist’s perspectives of device usability. | | Existing combination aid users with tinnitus and hearing loss. | Set of recommendations for conducting a randomised controlled trial of effectiveness of combination aids is formulated. | |
| 4 | Barozzi et al., 2016 | A comparison of nature and technical sounds for tinnitus therapy | Group 1: Sounds of nature: 19  Group 2: Broadband noise: 17 | Randomised Controlled Trial | Tinnitus Handicap Inventory,  Numeric Rating Scale (Annoyance) | | Tinnitus duration for at least 6 months. | Statistically significant improvement in THI scores (mean improvement of 10.96 points) in both groups (nature sounds streamed to hearing aids from mobile phone and broadband noise). No significant difference in improvement between the two groups. | |
| 5 | Berberian et al., 2016 | Benefit of Using the Prosthesis with Sound Generators in Individuals with Tinnitus Associated With Mild to Moderately Severe Hearing Loss | 25 | Uncontrolled before and after study – analysis of a subset of data | Pure tone audiometry with and without hearing aids; Visual Analogue Scale (annoyance), Tinnitus Handicap Inventory | | Mild to moderate hearing loss and bilateral tinnitus. | Statistically significant reduction in both VAS annoyance and THI scores after using combination aids. | |
| 6 | Searchfield et al., 2016* | Spatial masking: Development and testing of a new tinnitus assistive technology | 14 | Cross-over trial: proof-of-concept | Tinnitus Handicap Inventory | | Bilateral no greater than moderate hearing loss, continuous tinnitus. | Evaluation of a prototype in which spatial (3D) masking paradigm was compared to a bilaterally equal masker delivered using iPods connected to hearing aids. Participants preferred 3D masking to bilaterally equal masking. There were no differences between groups on the THI score. | |
| 7 | Williams & Patel, 2016 | An update on hearing aid technology | NA | Literature review: Describes different applications of combination aids for tinnitus and options available on the devices. Describes wireless streaming as one of the available options | NA | | Not specified | A low number of small scale studies have suggested that combination aids and wireless streaming might be useful for management of tinnitus with appropriate support/counselling. Larger controlled studies of efficacy are required. | |
| 8 | Henry et al. 2015 | Validation of a novel combination hearing aid and tinnitus therapy device | 30 (15 fitted with amplification and 15 with combination aids) | Prospective-Randomized Controlled Trial | Tinnitus Functional Index, Hearing Handicap Inventory for the Elderly | | Perceived hearing difficulties, no hearing aid experience within 12 months, symmetrical sensorineural hearing loss within mild to moderately severe range (4 frequency PTA: 25-70 dB HL). | Both groups (amplification only and combination hearing aids) revealed significant improvement after 3 months as measured with Tinnitus Functional Index. Difference between two groups at 3 months was not statistically significant. | |
| 9 | Langguth, 2015 | Treatment of tinnitus | NA | Literature review: Combination aids mentioned as a management option. | NA | | NA | Concludes even if sound stimulation is widely established, evidence for its efficacy based on controlled studies is still insufficient. | |
| 10 | Newman & Sandridge, 2015 | Sound Therapy Option Profile (STOP): A tool for selecting devices used in tinnitus treatment |  | STOP Assessment tool description | NA | | Those with a need of improving communication function. If the patient's hearing loss has no impact on the patients psychosocial and/or communication function (HHIE/HHIA; e.g.; 18 point), the audiologist should consider combination units. Motivation to use sound therapy. Acceptability of different sound options. Willingness to try ear-level devices. Previous experience with devices. | STOP was developed to be a brief and easy to complete tool that serves as a springboard for patient counselling while providing guidance in selection of specific sound generator devices. | |
| 11 | Piskosz & Dyrlund, 2015 | Flexible and personalized sound therapy | NA | Concept/Product description | NA | | Those who prefer other sounds over the broadband noise. Tinnitus and hearing loss. | Concludes wireless streaming allows personalization of sound therapy. | |
| 12 | Hicks et al. 2014 | Clinical validation of a novel combination hearing aid and tinnitus therapy device | 30 (15 combination aids; 15 amplification only) | Prospective-Randomized Controlled Trial (Conference abstract) | Tinnitus Functional Index | | Bothersome tinnitus, hearing aid candidate. | Both hearing aids and combination aids provided significant benefit for alleviating the effects of tinnitus. Experimental group showed a mean reduction in the TFI that was 8.6 points greater than the control group. There were no significant differences between groups. | |
| 13 | Hoare  et al. 2014 | Sound therapy for tinnitus management: practicable options | NA | Literature review | NA | | Bothersome tinnitus with or without hearing loss | Concludes sound therapy on its own is of unproven benefit; equally, there is little to suggest it is of potential harm to patients. Limited evidence of the benefit of sound therapy independent from other concomitant factors such as counselling. | |
| 14 | Johansen et al. 2014 | Effect of counselling, amplification and fractal tones in tinnitus management | 35 | Prospective-Uncontrolled Before and After Study | Tinnitus Handicap Inventory, a hearing aid and the Zen questionnaire to investigate overall satisfaction with the hearing aids and the relaxation effects of Zen, use time for different programs assessed | | Clinically significant tinnitus and hearing loss. No use of hearing aids for the previous 12months. | Statistically significant improvements in Tinnitus Handicap Inventory scores from baseline at 2 months counselling, 2 months post-amplification, 2 months post-fractal tones and 12 months post whole treatment (all in comparison to baseline). Post-counselling, post-Zen fit- significant improvements between phases, no improvement post-hearing aid fitting. | |
| 15 | Kong et al. 2014 | Frequencies characteristics of tinnitus and its impact on different sound-related treatment methods | 85 | Prospective-Uncontrolled Before and After Study (Conference abstract) | Not reported | | Unclear (poor performance in the Residual Inhibition test mentioned as well as high-frequency hearing loss to offer Zen sounds). | The Zen sound hearing aids gave a better life quality in a proportion of patients. Patients with normal hearing threshold and light hearing loss get more positive reaction than the severe hearing loss group. | |
| 16 | dos Santos et al., 2014 | The influence of sound generator associated with conventional amplification for tinnitus control: randomized blind clinical trial | 47 (24 combination aids and 23 amplification only) | Prospective-Randomized Controlled Trial | Tinnitus Handicap Inventory, numerical scales, psychoacoustic measures | | Mild to moderate bilateral symmetrical sensorineural hearing loss, complaints of constant tinnitus for at least 6 months, THI score more than 20. No previous experience with amplification. | No difference between THI scores between amplification only group and combination devices group after the intervention. Both groups showed reduction in THI scores after fitting. 62.5% (n=15) of combination aids users and 78% of amplification only users showed reduction of 20 or more in the THI. | |
| 17 | Toth et al., 2014 | Tinnitus Therapy | NA | Literature review | NA | | High-pitched tinnitus. | Concludes there is no strong evidence that change in loudness of tinnitus or the overall severity of tinnitus can be achieved by use of sound generating devices. | |
| 18 | Tunkel et al., 2014 | Clinical Practice Guideline: Tinnitus | NA | Systematic review /Practice guidelines | NA | | Hearing loss and tinnitus. | Concludes clinicians should consider sound therapy as an option for patients with persistent, bothersome tinnitus. | |
| 19 | Hall, 2013 | Siemens Expert Series: Evidence-based management of troublesome tinnitus – Practical guidelines for the practicing professional | NA | Guide: Combination aids mentioned as a management option | NA | | Tinnitus interfering with daily life despite amplification | None specific to combination aids | |
| 20 | Smith et al., 2013 | Complementary and integrative treatments: Tinnitus | NA | Literature review: Combination aids and different noise options available mentioned as a management option. | NA | | Not specified. | None specific to combination aids | |
| 21 | Sweetow, 2013 | The use of fractal tones in tinnitus patient management | NA | Literature review | NA | | Not specified | Concludes both fractal tones and white noise reduced tinnitus annoyance but the fractal tones were preferred by subjects for longer term use. | |
| 22 | Fioretti  et al. 2012 | Suppression of tinnitus in a patient with unilateral sudden hearing loss: a case report | 1 | Case report | Tinnitus Handicap Inventory, ULLs, Visual Analogue Scales (loudness, annoyance) | | Disabling tinnitus, sudden unilateral hearing loss. | The THI was significantly reduced at the follow-up evaluation after 6 months (THI 20; VAS 3) and 12 months (THI 14; VAS 2). Initial THI 80; VAS 9. | |
| 23 | Haberle & Hoejgaard, 2012 | Tinnitus treatment options in hearing aids: A novel approach to turning non-users into users | About 200 | Uncontrolled Before and After Study | Questionnaire on participants’ experiences and satisfaction with Zen feature and the CLEAR hearing aid | | Hearing impaired people with tinnitus who wanted to try hearing aids. | Majority of participants were satisfied with aids once persuaded to try them. Emphasizing the tinnitus treatment possibilities of hearing aids may be a novel avenue to persuading hearing-impaired non-owners with tinnitus to seek treatment. | |
| 24 | Hobson et al. 2012 | Sound therapy (masking) in the management of tinnitus in adults (Review) | NA | Cochrane Systematic Review | NA | | Depending on the study | Concludes there is no evidence that a significant change in loudness of tinnitus or the overall severity of tinnitus can be achieved following the use of sound-generating (masking) devices as a sole intervention. | |
| 25 | Piskosz, 2012* | The role of wireless streaming in tinnitus management | NA | Concept/Product description: Wireless streaming described as a flexible option that can address needs of any tinnitus management plan | | NA | Tinnitus and hearing loss. Those patients who prefer other noise types over the white noise. | | None specific to combination aids |
| 26 | dos Santos et al. 2012 | The use of hearing aids generic sound generator with integrated control of tinnitus- pilot study | 6 (3 fitted with hearing aid plus sound generator; 3 with amplification only) | Prospective-Pilot study (conference abstract) | Not reported | | Tinnitus and mild to moderate hearing loss | No changes in the psychoacoustic characteristics of tinnitus. However, there is less of tinnitus and hearing handicap in both groups, with combination aids group showing greater reduction of the handicap caused by the tinnitus than the amplification only group after 4 months. | |
| 27 | Hoare  et al. 2011 | Systematic review and meta-analyses of Randomized Controlled Trials examining tinnitus management. | NA | Systematic review | NA | | Not reported | Conclude sound enrichment interventions and outcome measures were not sufficiently similar to perform meta-analysis.  The efficacy of most interventions for tinnitus benefit remains to be demonstrated conclusively. | |
| 28 | Piskosz & Kulkarni, 2010* | An innovative combination device to assist in tinnitus management | 24 | Prospective-Uncontrolled Before and After Study | Tinnitus Handicap Inventory, Tinnitus Handicap Questionnaire | | Tinnitus and mild to moderate hearing loss. | Significant improvement in tinnitus questionnaire scores over 6 months | |
| 29 | Sweetow & Sabes 2010 | Effects of Acoustical Stimuli Delivered through Hearing Aids on Tinnitus | 16 | Prospective-Uncontrolled Before and After Study | Tinnitus Handicap Inventory, Tinnitus Reaction Questionnaire, relaxation ratings (1 very relaxing, 5 very tensing),Tinnitus Annoyance Scale (0-6) | | Primary complaint of tinnitus, sufficient hearing loss to warrant the use of amplification. | Significant decrease in the THI and TRQ scores after 6 months. Clinically significant improvement (THI, 20-point change) in 6 out of 14 participants. | |
| 30 | McFerran, 2009 | Tinnitus and hyperacusis | NA | Literature review: Combination aids mentioned as a management option | NA | | Tinnitus and hearing loss. | Concludes devices are complicated and difficult to set up satisfactorily | |
| 31 | Del Bo & Ambrosetti, 2007 | Hearing aids for the treatment of tinnitus | NA | Literature review: Combination aids described as a management option | NA | | Tinnitus and hearing loss. | Patients with hearing loss and tinnitus can obtain additional benefit from sound therapy by using combination aid. | |
| 32 | Henry et al., 2006a* | Clinical trial to compare tinnitus masking and tinnitus retraining therapy | 123 (42 used combination instruments in the tinnitus masking group and 13 in the TRT group) | Prospective-Quasi-randomized Controlled Study: Combination aids used as a part of Tinnitus Masking | Tinnitus Handicap Inventory, Tinnitus Handicap Questionnaire, Tinnitus Severity Index | | Sufficiently severe tinnitus and motivation to comply with the treatment. | Combination aids subgroup was not analysed. | |
| 33 | Henry  et al. 2006b* | Outcomes of clinical trial: Tinnitus Masking versus Tinnitus Retraining Therapy | 118 (Initial fitting: 39 combination aids in TM, 1 in the TRT; Final configuration-changes during treatment: 38 in TM and 12 in TRT) | Prospective-Quasi-randomized Controlled Study: Combination aids used as a part of Tinnitus Masking | Tinnitus Handicap Inventory, Tinnitus Handicap Questionnaire, Tinnitus Severity Index, percentage ratings of awareness of tinnitus (AWARE) and annoyance by tinnitus (ANNOY) | | The patient chose the device that they felt was the most effective and acceptable in providing some level of relief with the help of the clinician. | Combination aids subgroup was not analysed. | |
| 34 | McFerran & Phillips, 2006* | Tinnitus | NA | Literature review: Combination aids listed as one of the methods providing wearable sound therapy alongside hearing aids and noise generators | NA | | Not specified | NA | |
| 35 | Henry et al., 2005* | Clinical guide for audiologic tinnitus management II: Treatment | NA | Guide: Combination aids described as one of the sound therapy options | NA | | Hearing loss and problematic tinnitus. | Recommends to inform patients of the different uses of the ear-level devices and mutually establish device-usage protocol that best meets patient’s treatment needs. | |
| 36 | Henry et al., 2005* | General review of tinnitus: Prevalence, mechanisms, effects, and management | NA | Literature review: Combination aids mentioned as a management option. | NA | | Not specified | The primary purpose of either the combination aids used with TM and TRT is to treat tinnitus. Improvement in hearing is considered of secondary benefit. | |
| 37 | Henry et al., 2005* | Clinical management of tinnitus using ‘progressive intervention’ approach | NA | Literature review/Guide: Combination aids described as a management option within TM | NA | | Tinnitus and hearing loss. | None specific to combination aids | |
| 38 | Frachet et al., 2004 | Acoustic hearing aid with an integrated noise generator in hearing-impaired subjects with tinnitus | 28 | Prospective-Crossover Before and After Study | Visual Analogue Scale of tinnitus severity (SEV), Visual Analogue Scale of tinnitus distress (DET), Tinnitus Handicap Questionnaire | | Patient-reported debilitating tinnitus for at least 1 year, associated with a hearing loss of any duration of time. | Statistically significant lowering of scores for DET. THQ and SEV no statistically significant differences. Amplification only: worsening of TI (+8%), amplification plus sound - improvement (-10%). | |
| 39 | Lopez-Gonzalez and Lopez-Fernandez 2004 | Sequential sound therapy in tinnitus | 26 (SST; out of 38 devices 34 were combination aids) and 15 (TRT; all 30 devices were combination aids) | Prospective/Retrospective-Historically Controlled Trial | Tinnitus Handicap Inventory | | Not specified | Improvement in THI in 100% of patients treated with SST (no numerical data available), and in 33% of TRT patients (53% left the study). The perception of tinnitus diminished in all patients treated with SST (by 6 months or earlier). | |
| 40 | Vernon & Meikle, 2003 | Masking devices and alprazolam treatment for tinnitus | NA | Literature review/Guide: Combination aids described as a management option | NA | | High-pitched tinnitus and high-frequency hearing loss (above 3-4 kHz). Advise trying amplification first. | None specific to combination aids | |
| 41 | Schechter & Henry, 2002* | Assessment and treatment of tinnitus patients using a “masking approach” | NA | Guide | NA | | If hearing aids provide no masking benefit, then a tinnitus instrument may be tried, permitting the combined use of amplification and masking. | Concludes one of the minimal essential components of tinnitus management is trial use of ear-level devices (hearing aids, combination aids, or tinnitus maskers). | |
| 42 | Henry et al., 2002* | Comparison of tinnitus masking and tinnitus retraining therapy | NA | Literature review: Combination aids described as management option within TRT and TM | NA | | Selected by the patient through a trial-and-error procedure that is facilitated by the clinician. | None specific to combination aids | |
| 43 | Schechter et al., 2002* | Selection of ear level devices for two different methods on tinnitus treatment | 52 patients in the Masking group and 21 in the TRT group | Randomised Controlled Study | Patient preference for device | | Trial of the devices. | 38 out of 52 patients in the TM group (choice based on the trial of the instruments) received combination aids. | |
| 44 | Gabriels, 2001 | What the practitioner should know about tinnitus assessment and management | NA | Guide | NA | | Clinically significant tinnitus and hearing loss. | New digital hearing aids and combination sound generators/hearing aids offer the most flexibility and options when fitting the tinnitus patient | |
| 45 | Rosanowski et al., 2001 | Interdisciplinary management of chronic tinnitus (II) | NA | Literature review: Combination aids mentioned as a management option | NA | | Second choice if the hearing aids alone do not provide sufficient tinnitus masking. | Low efficacy and poor cost-effectiveness is postulated | |
| 46 | Folmer, 2000 | Managing chronic tinnitus as phantom audiotory pain | NA | Investigational study/Survey: Combination aids mentioned as a management option | Tinnitus Severity Index, abbreviated Becks Depression Inventory, State-Trait Anxiety Inventory | | Bothersome tinnitus | None specific to combination aids | |
| 47 | Vernon & Meikle, 2000 | Tinnitus Masking | NA | Literature review: Combination aids described as a management option including candidacy, testing routine, instructions to patients, clinical examples, bilateral vs unilateral masking and tinnitus relief during sleep. | NA | | When hearing aids are not effective enough for management of tinnitus. | None specific to combination aids | |
| 48 | Dobie 1999 | A review of Randomized Clinical Trials in Tinnitus | NA | Literature review | NA | | Not specified | Conclude none of the treatments studied (including combination aids) shown to eliminate tinnitus more frequently than placebo, or to provide replicable long-term effects. | |
| 49 | Peifer et al., 1999 | Tinnitus: Etiology and management | NA | Literature review | NA | | Minimal masking level of less than 10 dB | Concludes the tinnitus instrument appears to be the most successful of the three types of masking devices. It has been shown that as many as 55% of patients with tinnitus benefit from tinnitus instrument if they are capable of using it. | |
| 50 | Sandlin & Olsson, 1999 | Evaluation and selection of maskers and other devices used in the treatment of tinnitus and hyperacusis | NA | Literature review | NA | | Please see Johnson et al, 1989. | Concludes no [then] current approach to sound therapy is superior | |
| 51 | Goldstein & Shulman 1996 | Tinnitus Masking- A longitudinal study of efficacy/diagnosis 1977-1994 | 1888 (799 advised instrumentation) | Uncontrolled before and after study | Number of participants purchasing the device | | Medical Audiologic Tinnitus Patient Protocol (MATPP). Most important: maskability. | Of the 356 patients who purchased devices, 148 (42%) were tinnitus maskers, 67 (19%) were tinnitus instruments, 79 (22%) were hearing aids, and 62 (17%) were binaural fittings of various combinations of the three devices. | |
| 52 | O’Connor & Zappia, 1993 | Management of the tinnitus patient | NA | Practice description/Case studies: Combination aids mentioned as a management option | NA | | Tinnitus and hearing loss. | None specific to combination aids | |
| 53 | Goldstein & Shulman 1992 | Tinnitus Masking: A longitudinal study of efficacy/diagnosis 1977-1990 | 1574 (59 purchased tinnitus instrument after the trial; not stated how many were advised combination aid) | Uncontrolled Before and After Study | Number of participants purchasing the device | | Tinnitus and significant hearing loss. | Results not specific to tinnitus instruments. 59 out of 330 patients who purchased the instruments after trial period purchased tinnitus instruments.  Please note those results are also reported in Goldstein and Shulman, 1996 | |
| 54 | Tyler et al., 1992 | Recent advances in tinnitus | NA | Literature review: Combination aids mentioned as a management option | NA | | Tinnitus and hearing loss. | None specific to combination aids | |
| 55 | Hazell, 1990 | Tinnitus III: The practical management of sensorineural tinnitus | NA | Literature review: Combination aids mentioned as a management option | NA | | Tinnitus and hearing loss. | None specific to combination aids | |
| 56 | Johnson et al. 1989 | A Tinnitus Masking Program: Efficacy and safety | 370 (236 tried combination aids) | Uncontrolled Before and After Study | Number of participants purchasing the device | | Tinnitus and hearing loss. Also those having difficulty sleeping | The largest group of patients received recommendations for tinnitus instruments. This pattern has remained consistent over time. | |
| 57 | Hazell  et al. 1985 | A clinical study of tinnitus maskers | 472 (382 reaching the first evaluation session and 206 reaching the second evaluation session; only part fitted with combination instrument) | Prospective-design differed depending on the center: prospective controlled study, randomized controlled study | Masker Effectiveness Questionnaire, Crown Crisp Experimental Index, and Semantic differential scores | | Tinnitus and hearing loss. | Tinnitus maskers and combination instruments should be regarded as a useful treatment for patients suffering from tinnitus. Combination aids were less effective than maskers, and not much better than hearing aids. | |
| 58 | Stephens and Corcoran 1985 | A controlled study of tinnitus masking | 147 (5 groups, only one fitted with combination aids) | Prospective-Randomized Sub-study within Hazell et al. 1985 | Masker Effectiveness Questionnaire, Crown Crisp Experimental Index, and Semantic differential scores | | Tinnitus as a main complaint and hearing loss. | The differences between those fitted with sound generators, combination aids and amplification were small. | |
| 59 | Mehlum et al. 1984 | Prospective crossover evaluation of four methods of clinical management of tinnitus | 34 (trialled 4 management options) | Prospective-crossover before and after study | Diary, interview | | Not specified | No method was found more efficacious than another. | |
| 60 | Schleuning et al. 1980 | Evaluation of the Tinnitus Masking Program: a Follow-up Study of 598 Patients | 380 (44 tried combination aids) | Uncontrolled before and after study | Number of devices purchased, patients’ reports | | Tinnitus and hearing loss | None specific to combination aids | |
