# Supplementary material for: Combined Amplification and Sound Generation for Tinnitus: A Scoping Review
Source: Ear Hear. 2018 Apr 27;39(3):412–22. doi: 10.1097/AUD.0000000000000516 (PMC7664457; doi:10.1097/AUD.0000000000000516)
Supplement: Supplementary file 2 [file aud-39-412-s002.docx]

| Table. 2. Charting the data for studies where sound therapy is used in the context of comprehensive intervention strategy (i.e. in conjunction with psychological therapy). Records marked with * present data regarding sound therapy as a main component of the intervention (see Table.1) as well as comprehensive intervention strategy. | | | | | | | | | | | | |
| --- | --- | --- | --- | --- | --- | --- | --- | --- | --- | --- | --- | --- |
| **No** | **Authors, Year** | **Title** | | | **Participants** | | **Type of study** | | **Measures** | **Candidacy** | | **Main finding/ Conclusion** |
| 1 | Ibarra et al. 2017* | In-ear medical devices for acoustic therapies in tinnitus treatments, state of the art | | | NA | | Literature review | | NA | Not specified | | Combination aids mentioned as a management option within Widex Zen Therapy. |
| 2 | Stocking & Stecker, 2017 | Efficacy of the Individual Components of a Tinnitus Management Protocol | | | 17 | | Uncontrolled before and after study | | Visual Analogue Scales (severity, awareness, annoyance, general level of relaxation, sensitivity to loud sound), Tinnitus Functional Index, Minimum Masking Level, Loudness Discomfort Level, questionnaire on perceived overall benefit of each Widex Zen Therapy component. | Tinnitus with or without hearing loss (without hearing loss programmed with minimal or no amplification). | | Widex Zen Protocol used was found to be effective in reducing TFI scores (from 62 to 34) and VAS annoyance and awareness. Over 12 months increase in use of amplification only programme and decrease in use of Zen tones was observed. |
| 3 | Tarnowska et al., 2017 | Tinnitus treatment as a problem area | | | NA | | Literature review: Combination aids mentioned as a predominant device used in management of TRT Category 2 patients | | NA | TRT Category 2. | | None specific to combination aids |
| 4 | Bauer et al., 2016 | Clinical trials supported by the Tinnitus Research Consortium: Lessons learned, the Southern Illinois University experience | | | NA | | Review of clinical trials supported by the Tinnitus Research Consortium: describes an ongoing quasi-radomized trial designed to assess and quantify TRT treatment effects. Participants were fitted with Resound combination device | | NA | TRT Category 2. | | None specific to combination aids |
| 5 | Searchfield et al., 2016* | Spatial masking: Development and testing of a new tinnitus assistive technology | | | 9 | | Cross-over pilot study of a method for the spatial (3D) masking of tinnitus. Combined amplification and 3D masking was compared to TRT using a conventional 2D sound within a combination aid | | Tinnitus Functional Index | Bilateral no greater than moderate hearing loss, continuous tinnitus. | | Participants preferred 3D masking to 2D masking. There were no difference between groups on the TFI score. |
| 6 | Powers and dos Santos., 2015 | Acoustic therapy for the treatment of tinnitus: A primer | | | NA | | Guide: Describes setting of the therapy signal according to Tinnitus Retraining Therapy and Tinnitus Activities Treatment | | NA | Tinnitus and hearing loss if the amplification is sufficient in reducing patient’s reaction to tinnitus. | | NA |
| 7 | Sweetow et al., 2015 | Do tinnitus patients continue to use amplification and sound therapy post habilitation? | | | 19 | | Prospective-Uncontrolled Before and After Study: Combination aids used as a part of Widex Zen Therapy | | Tinnitus Handicap Inventory, Tinnitus Functional Index, Widex Tinnitus Intake Questionnaire | Tinnitus and hearing loss. | | Mean improvement in Tinnitus Functional Index score was 48 points. Significant improvement was noted between baseline and 2 months (largest change in the TFI scores) as well as 4 and 6 months post fitting. |
| 8 | Epstein, 2014 | Tinnitus management fundamentals: Widex Zen Therapy | | | NA | | Concept/Product description: Guide on the use of Zen fractal tones and Widex Zen Therapy | | NA | Tinnitus and hearing loss. | | NA |
| 9 | Herzfeld et al., 2014 | Clinical trial on the effectiveness of Widex Zen Therapy for tinnitus | | | 24 | | Prospective-Uncontrolled Before and After Study: Combination aids used as a part of Widex Zen Therapy. | | Tinnitus Functional Index, Tinnitus Handicap Inventory, Widex Tinnitus Intake Questionnaire | Clinically significant tinnitus. | | Significant reduction between baseline and 2 months in both Tinnitus Handicap Inventory and Tinnitus Functional Index scores. No further significant difference between 2 and 6 months. |
| 10 | Oz  et al. 2013 | | Effectiveness of the combined hearing and masking devices on the severity and perception of tinnitus: A randomized, controlled, double-blind study | 21 (12 betahistine dihydrochloride plus combination aid or a sound generator; 9 betahistine 2HCl) | | Prospective-Randomized Controlled Trial | | Visual Analogue Scale, Mini-Tinnitus Questionnaire | | Primary complaint of tinnitus and hearing loss. | Tinnitus loudness did not differ before and after treatment. VAS ratings were significantly reduced before and after treatment. Significant difference before and after treatment for Mini-TQ. No significant difference between groups for any of the above measures. | |
| 11 | Sekiya et al., 2013 | Using fractal music as sound therapy in TRT treatment | | | 25 (out of 33 who chose fractal music as a sound therapy option, not clear if with amplification) | | Prospective-Uncontrolled Before and After Study: Combination aids with Zen tones used as a part of Tinnitus Retraining Therapy. | | Tinnitus Handicap Inventory, Visual Analogue Scale (loudness, frequency of occurrence), satisfaction with sound therapy, , pleasantness of musical stimuli tiredness of musical stimuli | Tinnitus and hearing loss. | | The average THI of combination aid users fell from 58.3 at the initial visit to 30.1 after 3 months, and at a further 28.0 after six months. |
| 12 | Butcher & Davies, 2012 | Tinnitus: diagnosis and management | | | NA | | Literature review: Combination aids mentioned as a treatment option available in a secondary care as part of the Tinnitus Retraining Therapy | | NA | Not specified. | | NA |
| 13 | Piskosz, 2012* | The role of wireless streaming in tinnitus management | | | NA | | Concept/Product description: Combination aids and wireless streaming option mentioned as an option that meets requirements of Tinnitus Retraining Therapy and Progressive Tinnitus Management. | | NA | Tinnitus and hearing loss. Those patients who prefer other noise types over the white noise. | | NA |
| 14 | Sweetow & Kragh Jeppesen, 2012 | A new integrated program for tinnitus patient management; Widex Zen Therapy | | | NA | | Guide on the use of Widex Zen Therapy. | | NA | Tinnitus and hearing loss. | | NA |
| 15 | Hoare et al., 2013 | Recent technological advances in sound-based approaches to tinnitus treatment: A review of efficacy considered against putative physiological mechanisms | | | NA | | Literature review: Combination aids used as a part of Widex Zen Therapy. | | NA | Not specified. | | Conclude that future evaluation should consider neurophysiological measures of limbic system activity to quantify the effect of listening to the fractal tones on the stress response. |
| 16 | Jastreboff & Jastreboff, 2010 | The role of hearing aids in management of tinnitus | | | NA | | Literature review: Combination aids described in the context of Tinnitus Retraining Therapy. | | NA | Not specified. | | Concludes that setting combination aids requires specific knowledge and extensive education. |
| 17 | Korres et al., 2010 | Tinnitus Retraining Therapy (TRT): outcomes after one-year treatment | | | 33 (Category 1 or 2/TRT; Category 2-29 fitted with COMBI devices, Category 1- noise generators-4); 30 (refused TRT, treated with vasoactive medications) | | Prospective Non-randomized Controlled Study: Combination aids used as a part of Tinnitus Retraining Therapy. | | Tinnitus Handicap Inventory, Visual Analogue Scales (annoyance during sleep, concentration, relaxation, work) | TRT Category 2. | | Combination aids subgroup was not analyzed. |
| 18 | Kuk et al., 2010 | The efficacy of fractal music employed in hearing aids for tinnitus management | | | 26 | | Prospective-Survey of clinicians: Combination aids used as a part of tinnitus management by clinicians experienced in treating tinnitus and trained in Tinnitus Retraining Therapy, Cognitive Behaviour Therapy or Neuromonics. | | Tinnitus Reaction Questionnaire | Not specified. | | 100% of the patients who completed the pre- and post-TRQ questionnaire reported a reduction in tinnitus. Zen could be an effective sound therapy tool. |
| 19 | Piskosz & Kulkarni, 2010* | An innovative combination device to assist in tinnitus management | | | 30 | | Prospective-Uncontrolled Before and After Study: Combination aids used as a part of Tinnitus Retraining Therapy. | | Study 1: Tinnitus Handicap Inventory, TRI Tinnitus Patient Assessment and Outcome Measurement, Structured Interview | TRT Category 1 and 2. | | Significant reduction of THI score at 6 months post treatment, significant reduction on VAS scales for annoyance, intensity and tinnitus effects on patient's life at 6 months. |
| 20 | Shulman & Goldstein, 2010 | Principles of tinnitology: Tinnitus diagnosis and treatment: A Tinnitus-targeted therapy | | | NA | | Guide/Literature review: Combination aids mentioned as a management option used as a part of Tinnitus-Targeted Therapy and Tinnitus Retraining Therapy | | NA | Tinnitus and hearing loss of mixed or predominantly sensorineural type. Instrumentation considered for those patients who are resistant to attempts of tinnitus relief. | | NA |
| 21 | Henry et al., 2008 | Using therapeutic sound with Progressive Audiologic Tinnitus Management | | | NA | | Literature review: Combination aids described as one of the sound therapy options within Progressive Audiologic Tinnitus Management and Tinnitus Retraining Therapy | | NA | TRT Category 1 and 2. | | None specific to combination aids |
| 22 | Newman et al., 2008 | Strategies for managing patients with tinnitus: A clinical pathway model | | | NA | | Clinical pathway description: Combination aids mentioned as a management option within multidisciplinary Tinnitus Management Clinic | | NA | **Not specified.** | | None specific to combination aids |
| 23 | Jastreboff, 2007 | Sound therapies for tinnitus management | | | NA | | Literature review: Combination aids described as a management option within Tinnitus Retraining Therapy. | | NA | TRT Category 2. | | None specific to combination aids |
| 24 | McFerran & Phillips, 2007* | Tinnitus | | | NA | | Literature review | | NA | Not specified. | | Combination aids listed as one of the methods providing wearable sound therapy alongside hearing aids and noise generators as part of Tinnitus Retraining Therapy. |
| 25 | Henry et al., 2006a* | Clinical trial to compare tinnitus masking and tinnitus retraining therapy | | | 123 (42/59 used combination instruments in the tinnitus masking group and 13/63 in the TRT group) | | Prospective-Quasi-randomized Controlled Study: Combination aids used as a part of Tinnitus Retraining Therapy. Combination aids subgroup was not analysed | | Tinnitus Handicap Inventory, Tinnitus Handicap Questionnaire, Tinnitus Severity Index | TRT Category 2. | | None specific to combination aids |
| 26 | Henry et al., 2006b* | Outcomes of clinical trial: Tinnitus Masking versus Tinnitus Retraining Therapy | | | 118 (Initial fitting: 39/59 combination aids in TM, 1/64 in the TRT; Final configuration-changes during treatment: 38/59 in TM and 12/64 in TRT) | | Prospective-Quasi-randomized Controlled Study: Combination aids used as a part of Tinnitus Retraining Therapy. Combination aids subgroup was not analysed | | Tinnitus Handicap Inventory, Tinnitus Handicap Questionnaire, Tinnitus Severity Index, percentage ratings of awareness of tinnitus (AWARE) and annoyance by tinnitus (ANNOY) | TRT Category 2. | | None specific to combination aids |
| 27 | Jastreboff & Jastreboff, 2006 | Tinnitus Retraining Therapy: A different view on tinnitus | | | NA | | Literature review: Combination aids described as one of the sound therapy options within Tinnitus Retraining Therapy | | NA | Not specified. | | None specific to combination aids |
| 28 | Mazurek et al., 2006 | A modified version of tinnitus retraining therapy: Observing long-term outcome and predictors | | | 65 (mix of combination aids, amplification only, sound generators, 1.1% fitted with combination aids) | | Retrospective- Uncontrolled Before and After Study: Combination aids used as a part of modified Tinnitus Retraining Therapy | | Tinnitus Questionnaire, loudness | If hearing loss affected the speech audibility, the patients were supplied with hearing aid or combination aids. | | Combination aids subgroup was not analysed. |
| 29 | Henry et al., 2005* | Clinical management of tinnitus using a ‘progressive intervention’ approach | | | NA | | Literature review/Guide: Combination aids described as a management option within Tinnitus Retraining Therapy and Tinnitus Masking within the progressive-intervention approach | | NA | Not specified. | | None specific to combination aids |
| 30 | Henry et al., 2005* | Clinical guide for audiologic tinnitus management II: Treatment | | | NA | | Guide: Combination aids described as one of the sound therapy options within Audiologic Tinnitus Management in the context of Tinnitus Retraining Therapy | | NA | Patient and clinician mutually establish device-usage protocol | | None specific to combination aids |
| 31 | Henry et al., 2005* | General review of tinnitus: Prevalence, mechanisms, effects, and management | | | NA | | Literature review: Combination aids mentioned as a management option within Tinnitus Retraining Therapy | | NA | Not specified | | Concludes the primary purpose of either the combination aids or the hearing aids is to treat tinnitus. Improvement in hearing is considered of secondary benefit. |
| 32 | Jastreboff & Jastreboff, 2003 | Tinnitus Retraining Therapy for patients with tinnitus and decreased sound tolerance | | | NA | | Literature review: Combination aids described as one of the sound therapy options within Tinnitus Retraining Therapy | | NA | TRT Category 2 and 3. | | None specific to combination aids |
| 33 | Henry et al., 2002* | Comparison of Tinnitus Masking and Tinnitus Retraining Therapy | | | NA | | Literature review: Combination aids described as management option within Tinnitus Retraining Therapy | | NA | TRT Category 2. | | None specific to combination aids |
| 34 | Henry et al., 2002 | Assessment of patients for treatment with Tinnitus Retraining Therapy | | | NA | | Guide: Combination aids as an option within Tinnitus Retraining Therapy | | NA | TRT Category 2. | | None specific to combination aids |
| 35 | Schechter et al., 2002* | Selection of ear level devices for two different methods of tinnitus treatment | | | 52 patients in the Masking group and 21 in the TRT group | | Randomised Controlled Study: Combination aids used as a part of Tinnitus Retraining Therapy. | | Not reported | TRT Category 2. | | 38 patients in the Masking group and 4 in TRT group received combination devices |
| 36 | Von Wedel & von Wedel, 2000 | Tinnitus retraining therapy – an update | | | NA | | Literature review: Combination aids mentioned in the context of Tinnitus Retraining Therapy | | NA | Option for tinnitus with high-frequency hearing loss. | | Based on study by McKinney et al., 1999 conclude that devices are not necessary for the success of TRT. |
| 37 | McKinney et al., 1999 | An evaluation of the TRT method | | | 13 (out of 182 participants fitted with combination aids, the rest directive counselling only or counselling and sound generators or counselling and amplification only) | | Prospective- Non-randomized Controlled Study: Combination aids used as a part of Tinnitus Retraining Therapy | | Tinnitus questionnaire scales: annoyance, effect on life quality, loudness, % awareness, tinnitus pitch, Minimum Masking Levels | Tinnitus and moderate to severe hearing loss. | | No differences between groups using different types of devices. Significantly lower levels of annoyance, effect on life quality, loudness and percentage awareness after 12 months than at the start of the study in all patients. Directive counselling appears to be the most important element of TRT. The gain in wearing any form of instrument in addition to directive counselling appeared to be minimal. |
| 38 | Jastreboff et al., 1994 | Neurophysiological model of tinnitus: Dependence of the minimal masking level on treatment outcome | | | 74/365 patients were fitted with combination aids | | Sub study within Hazell et al., 1985 (See Supplemental Table 1) Combination aids used in the context of Tinnitus Retraining Therapy | | Minimal Masking Level, intensity match, threshold of hearing for wide band noise. | Not specified. | | The type of device used had no significant impact on the treatment outcome. |
